# Étude interventionnelle sur le dolutégravir et les autres antirétroviraux dans l’athérosclérosclérose infra-clinique en milieu hospitalier de Kinshasa

**DOI:** 10.11604/pamj.2023.45.63.39461

**Published:** 2023-05-26

**Authors:** Murielle Longokolo Mashi, Marcel Mbula Mambimbi, Hippolyte Nani-Tuma Situakibanza, Madone Mandina Ndona, Jean-Robert Makulo Risassi, Nadine Mayasi Ngongo, Ben Bepouka, Odio Ossam, Jean Mukaya Tshibola, Frédéric Tshibasu Tshienda, Eric Mukenge Kasongo, Mamy Ngole Nzita, Lukiana Tuna, Aimée Lulebo, Donatien Mangala Sonzi, Christian Kisoka Lusunsi, Benjamin Longo-Mbenza

**Affiliations:** 1 Service des Maladies infectieuses, Cliniques Universitaires de Kinshasa, Kinshasa, République Démocratique du Congo,; 2 Service de Néphrologie, Cliniques Universitaires de Kinshasa, Kinshasa, République Démocratique du Congo,; 3 Département de Radiologie, Cliniques Universitaires de Kinshasa, Kinshasa, République Démocratique du Congo,; 4 Service de Biologie Clinique, Cliniques Universitaires de Kinshasa, Kinshasa, République Démocratique du Congo,; 5 École de Santé Publique, Université de Kinshasa, République Démocratique du Congo,; 6 Department of Public Health, Lomo University of Research, Kinshasa, Democratic Republic of Congo,; 7 Service de Cardiologie, Cliniques Universitaires de Kinshasa, Kinshasa, République Démocratique du Congo,; 8 Walter Sisulu University, Department of Health, Mthatha, South Africa

**Keywords:** Athérosclérose infraclinique, VIH/SIDA, thérapie antirétrovirale, Kinshasa, République Démocratique du Congo, Subclinical atherosclerosis, HIV/AIDS, antiretroviral therapy, Kinshasa, Democratic Republic of Congo

## Abstract

**Introduction:**

après 2016, l´Organisation mondiale de la Santé (OMS) a proposé le dolutégravir (DTG) comme alternative thérapeutique de traitement de première ligne chez l´adulte. Ainsi, l´objectif de la présente étude était d´identifier les biomarqueurs du risque cardiométabolique capable de démontrer l´effet bénéfique du dolutégravir (DTG) par rapport aux autres antirétroviraux dans la prédiction de l´athérosclérose chez les personnes vivant avec le VIH (PVVIH) en milieu hospitalier de Kinshasa.

**Méthodes:**

il s´agissait d´une étude interventionnelle entre janvier 2017 et décembre 2021 chez les PVVIH sous traitement antiretroviral (TAR) durant au moins 6 mois, pris en charge dans les structures du Réseau Catholique du Bureau Diocésain des Oeuvres Médicales (BDOM) et aux Cliniques Universitaires de Kinshasa (CUK). L´athérosclérose infraclinique était définie par: une pression pulsée (PP) ≥60 mm Hg; une épaisseur intima-media carotidien (EIMc)> 0,8 mm et un Index des pressions systoliques (IPS) <0,9. La régression logistique a été utilisée dans l'étude statistique des associations.

**Résultats:**

au total, 334 PVVIH ont été recrutées dont 96,1% (n=321) sous TAR et 13,9% (n=13) naïfs de TAR. L´âge moyen des PVVIH était de 51±12 ans avec une prédominance féminine 70,4% (n=235); Les déterminants indépendants de l´athérosclérose infraclinique étaient les mariés (ORa: 4, IC95% 1,5-10,5; p<0,006), le niveau socioéconomique bas (ORa: 10,7, IC95% 2,3-48,7 p<0,002), la durée de l´infection par le VIH (ORa: 6,6, IC95% 2,8-16; p<0,0001), la durée du traitement antirétroviral ≥9 années (ORa: 0,3, IC 95% 0,2-0,7; p< 0,005) et le ratio cholestérol total/high density lipoprotein-cholesterol (CT/HDL-c)(ORa: 2, IC 95% 1,1-3,6; p=0,034). Les valeurs moyennes des variables traditionnelles et émergentes étaient significativement supérieures dans l´ancien régime TAR sans DTG que dans le nouveau régime avec DTG. Par contre la dyslipidémie a été identifiée du côté du nouveau régime avec DTG.

**Conclusion:**

la dyslipidémie a été fréquente du côté de DTG. Les mariés, le niveau socioéconomique bas, la durée de l´infection par le VIH, la durée du traitement antirétroviral au-delà de 9 années et le ratio CT/HDL-c ont été identifiés comme déterminants de l´athérosclérose infraclinique chez les PVVIH sous TAR en milieu hospitalier de Kinshasa.

## Abstract (en)

**Introduction:**

After 2016, the World Health Organization (WHO) proposed Dolutegravir (DTG) as an alternative first-line treatment for adults. Thus, the purpose of this study was to identify biomarkers of cardiometabolic risk capable of demonstrating the beneficial effect of Dolutegravir (DTG) compared to other antiretrovirals in predicting atherosclerosis in people living with HIV (PLHIV) and hospitalized in Kinshasa Hospital.

**Methods:**

we conducted an interventional study of people living with HIV who had received antiretroviral therapy (ART) for at least 6 months and were treated in the structures of the network coordinated by the Catholic Church (BDOM-Bureau Diocésain des Oeuvres Médicales) and of the University Clinics of Kinshasa (CUK) between January 2017 and December 2021. Subclinical atherosclerosis was defined as Pulsed Pressure (PP) ≥60 mm Hg; Carotid Intima-Media Thickness (CIMT) > 0.8 mm; and Systolic Pressure Index (SPI) < 0.9. Logistic regression was used in the statistical analysis of associations.

**Results:**

a total of 334 PLHIV were recruited, of whom 96.1% (n=321) were on ART and 13.9% (n=13) were ART naïve patients. The mean age of PLHIV was 51±12 years with a female predominance (70.4%; n=235); the independent determinants of subclinical atherosclerosis were marital status (aOR: 4. 95% CI 1.5-10.5; p<0.006), low socioeconomic level (aOR: 10.7, 95% CI 2.3-48.7 p<0.002), duration of HIV infection (aOR: 6.6, 95% CI 2.8-16; p<0.0001), duration of antiretroviral therapy ≥9 years (aOR: 0.3, 95% CI 0.2-0.7; p<0.005) and total cholesterol ratio/high-density lipoprotein-cholesterol (CT/HDL-c)(aOR: 2, 95% CI 1.1-3.6; p= 0.034). The mean values of traditional and emergent variables were significantly higher in the previous ART regimen without DTG than in the new regimen with DTG. However, dyslipidemia was detected during the new DTG-based regimen.

**Conclusion:**

dyslipidemia was common during the DTG-based regimen. Marital status, low socioeconomic level, duration of HIV infection, duration of antiretroviral treatment beyond 9 years and the TC/HDL-c ratio were identified as determinants of subclinical atherosclerosis in PLHIV on ART hospitalized in the Kinshasa hospital.

## Introduction

Après 2016, l´Organisation mondiale de la Santé (OMS) a proposé le dolutégravir (DTG) comme alternative thérapeutique de traitement de première ligne chez l´adulte [1,2]. Cette alternative thérapeutique repose sur des preuves scientifiques autour des complications cardiométaboliques liées aux anciennes molécules (Inhibiteurs Non Nucléosidiques de la Transcriptase) [3-5]. Ces complications cardiométaboliques favorisent l´athérosclérose infra-clinique [3-7] et l´athérosclérose clinique ou manifeste [3-6].

Le Programme National de Lutte contre le VIH/SIDA (PNLS) en République Démocratique du Congo (RDC) a adopté le Traitement antirétroviral (TAR) à base de dolutégravir comme traitement de première intention. La combinaison ténofovir (TDF)/lamivudine(3TC)/dolutégravir (DTG) soit TLD à dose fixe devra être introduite progressivement avec les nouveaux patients éligibles, en même temps le remplacement des anciens schémas par le nouveau schéma (TLD) pour ceux qui sont éligibles devra être fait graduellement [8]. Ainsi, l´objectif de la présente étude était d´identifier les biomarqueurs du risque cardiométabolique capable de démontrer l´effet bénéfique du dolutégravir (DTG) par rapport aux autres antirétroviraux dans la prédiction de l´athérosclérose chez les PVVIH en milieu hospitalier de Kinshasa.

## Méthodes

**Conception et cadre de l´étude:** il s´agit d´une étude interventionnelle réalisée dans les structures du réseau catholique du Bureau Diocésain des œuvres Médicales (BDOM) et aux Cliniques Universitaires de Kinshasa (CUK) entre janvier 2017 et décembre 2021. Le BDOM est un réseau qui regorge un plus grand échantillon des PVVIH dans la ville de Kinshasa. Par contre les Cliniques Universitaires de Kinshasa qui constituent un niveau tertiaire des soins, niveau de haute technicité était retenue comme cadre de référence.

**Population d´étude:** elle concernait les PVVIH âgées d´au-moins 18 ans naïves de TAR ou recevant un TAR depuis au-moins 6 mois et ayant donné librement son consentement; etaient exclues les PVVIH avec une grossesse, un syndrome néphrotique, une cirrhose hépatique, celles utilisant des médicaments hypolipémiants ou de l'insuline et celles ayant refusé de signer le consentement éclairé. La taille de l´échantillon a été calculée à partir de la formule de SCHWARTZ:


$$n=\frac{Z^{2}p\left(1-p\right)}{e^{2}}$$

La prévalence réelle de l´athérosclérose infra clinique chez les PVVIH en RDC étant inconnue, la prévalence de l´athérosclérose de 64,7% chez les PVVIH, rapportée par Aboubakar *et al*. [9] au Centre Hospitalier Universitaire de Treichville en Côte d´Ivoire a servi de base de référence. D´où la présente étude a retenu une fréquence de 65% pour le calcul de la taille minimale de l´échantillon.


$$n=\frac{1.96^{2}x0.65\left(1-0.65\right)}{0.05^{2}}=349.58$$

La taille d´échantillon calculée était de 350 sujets VIH+. La présente étude a classé deux groupes mutuellement exclusifs des patients âgés d´au-moins 18 ans infectés par le VIH sous TAR pendant au-moins 6 mois avec des critères prédéfinis: 1^er^ groupe était constitué des patients repartis selon les groupes des TAR: dolutégravir (DTG)+autres ARV, efavirenz (EFV)+autres ARV, névirapine (NVP)+autres ARV et lopinavir/ritonavir (LPV/r)+ autres ARV. Le deuxième groupe était constitué des patients sous ancien régime de TAR sans DTG et sous nouveau régime de TAR avec DTG.

**Collecte des données:** elle a été faite sur base d´une fiche ad hoc de collecte des données reprenant les paramètres démographiques, anthropométriques, cliniques et para cliniques. Les caractéristiques sociodémographiques comprenaient l´âge, le sexe et le niveau socioéconomique. Les données cliniques comprenaient les stades cliniques de l´infection par le VIH selon l´OMS, la durée de l´infection à VIH, la durée du traitement par le VIH et le régime thérapeutique. L'anthropométrie a enregistré le poids, la taille, le tour de taille (TT), le tour de hanches (TH), l'indice de masse corporelle (IMC = poids en kg/taille en m^2^) en utilisant des méthodes standard chez les participants avec des vêtements légers et sans chaussures, à l´aide d´une balance impédancemètre OMRON BF214 type BODY Composition Monitor, d´un mètre ruban et d´une toise. La pression artérielle (PA), y compris la pression artérielle systolique (PAS), la pression artérielle diastolique (PAD) et la pression pulsée (PAS-PAD) après que le participant se soit reposé pendant 10 minutes assis dans une pièce calme, a été mesurée dans le bras gauche avec le coude fléchi au niveau du cœur à l'aide d'un manomètre électronique Omron HEM 705 (Omron Life Science Co. Ltd, Tokyo, Japon). Les analyses de laboratoire comprenaient: la Protéine-C réactive (CRP), le glucose, le cholestérol total (TC), les triglycérides (TG), le High Density Lipoprotein-cholesterol (HDL-C), les tests sérologiques pour le VIH, l'acide urique et la créatinine sérique. Les valeurs de la charge virale datant de tout au plus 3 mois ont été recueillis dans les dossiers médicaux des participants. Toutes les analyses (hématologie et biochimie) ont été réalisées au laboratoire de Biochimie et d´Hématologie des Cliniques Universitaires de Kinshasa. Le test sérologique VIH a été réalisé sur chaque échantillon de sang (selon l´algorithme du Programme National de Lutte contre le VIH/Sida (PNLS)/RDC en vigueur pour confirmer la séropositivité des cas VIH inclus.

Les données d´imagerie médicale ont compris l´Index des pressions systoliques (IPS) ou Ankle Brachial Index (ABI) déterminées à l'aide d'une sonde 8 MHz à dispositif Doppler à onde continue, de marque HUNTLEIGH, tenue à la main comme décrit par Kwiatkowska *et al*. [10] et Olalla *et al*. [11] et la mesure de l´épaisseur intima-media carotidien (EIMc) réalisée à l´aide d´un appareil d´échographie Doppler équipé d´une sonde linéaire de 7,5-MHZ de marque PHILIPS. Le scannage était fait d´abord au niveau de la carotide commune droite puis la gauche. Les zones d´intérêt étaient définies comme une distance de 0,5 cm, 1 cm et 2 cm à partir de la bifurcation. Sur chaque zone d´intérêt, les épaisseurs murales proche et éloignée étaient mesurées; les valeurs maximales des mesures de l´Intima Media Thickness (IMT) étaient utilisées s´il existait une plaque d´athérome. La valeur de l´ABI (IPS) a été déterminée en divisant la pression la plus élevée des deux artères au niveau des chevilles par la pression artérielle systolique brachiale la plus élevée. En utilisant la formule ci-dessous, l'IPS a été calculé comme suit: IPS= PAS cheville/PAS bras. Avec PAS cheville (la pression systolique de l'artère tibiale postérieure ou de l'artère du dos du pied), et PAS bras (la pression systolique la plus élevée entre les deux membres supérieurs).

**Définitions de travail:** l´hypertension artérielle était définie par une PAS ≥ 140 mmHg et une PAD ≥90 mm Hg ou la prise courante d´antihypertenseurs [12,13]. L´indice de masse corporelle (IMC) était défini par le rapport poids exprimé en kg sur la taille en m^2^, l´obésité totale étant définie par une valeur > 30kg/m^2^ [14]. Le diabète sucré était défini par une glycémie à jeun ≥126 mg/dl ou la prise d´antidiabétiques [14]. L´augmentation du cholestérol HDL ≥ 75mg/dl était considérée comme facteur de risque cardiovasculaire [14,15]. L´athérosclérose sub-clinique (préclinique) était définie par: une pression pulsée ≥60 mmHg; une EIMc >0,8 mm et un ABI <0,9 [6,9,16,17]. Une charge virale supprimée était définie par un taux d´ARN-VIH plasmatique ≤1000 copies/mL et une charge virale indétectable était < 50copies/mL [18]. La Protéine-C Réactive (CRP)>3mg/L était considéré comme facteur de risque cardiovasculaire [19]. La maladie rénale chronique était définie par une ClCr < 60 ml/min en référence à la classification de NK-K/DOQI [20,21]. L´hyperuricémie était définie par une uricémie ≥ 7 mg/dL [22].

Le ratio Triglycérides/Glucose(TyG) était calculé par ln [TG (mg/dL)xFG(mg/dL)/2], permet de déterminer l´insulinorésistence, il est un biomarqueur plus efficace que ses composants séparés pour identifier les anomalies du métabolisme du glucose [23]. Sa valeur normale est fixée à 4,49. Le Non HDL-c était calculé par la différentielle entre le cholestérol total et le HDLc (CT-HDL-c) [24]. Chez les patients considérés à risque cardiovasculaire élevé, le taux de cholestérol non HDL est < 130 mg/dl. Chez les patients considérés à risque cardiovasculaire très élevé, ce taux est < 100 mg/dl. Le ratio CT/HDL-c permet de mesurer le risque cardiovasculaire [24,25]. Sa valeur normale doit être < 5. LDL/HDL coefficient athérogène était considéré comme prédicteur du risque cardiovasculaire si le rapport était >3,3 [26]; Le ratio TG/HDL-c ≥3 était considéré comme marqueur d´insulinorésistance [27]. Le ratio acide urique/HDL-c ≥10,9% était considéré comme un prédicteur du syndrome métabolique [28].

**Analyses statistiques:** les données ont été saisies à l´aide du logiciel Excel 2013, exportées et analysées à l´aide du logiciel IBM SPSS 26. Les analyses statistiques ont considéré une approche descriptive (moyenne±écart-type, fréquence, proportion), comparative univariée pour les facteurs associés et l´analyse multivariée de type régression logistique binaire pour identifier les déterminants indépendants prédisposant à l´athérosclérose infraclinique (variable dépendante) après ajustement pour les variables de confusion [Odds Ratio ajusté (ORa); Intervalle de Confiance à 95% (IC95%)]. Une valeur de P < 0.05 a été considérée comme statistiquement significative.

**Considérations éthiques:** tous les participants ont fourni un consentement éclairé écrit avant de participer à l'étude. Cependant, les dossiers/informations des patients ont été rendus anonymes et anonymisés avant l'analyse. Le protocole de cette étude a été soumis et approuvé par le comité éthique de l´école de Santé Publique de l´Université de Kinshasa selon le respect des recommandations d´Helsinki (N° App: ESP/CE/101/2020).

## Résultats

**Caractéristiques générales de la population d´étude:** parmi les 334 PVVIH de la population d´étude 96,1% (n=321) étaient sous traitement antirétroviral (TAR) contre 13,9% (n=13) naïfs de TAR; les femmes étaient majoritaires à 70,4% (n=235); l´âge moyen des patients était 51±12 ans avec des extrêmes de 20 ans et de 75 ans; le stade 3 de l´OMS était le plus représenté à 30,8% (n=103), les PVVIH sous efavirenz étaient majoritaires à 69,8% (n=224). Le Tableau 1 résume les caractéristiques générales de la population d´étude. Les données sont comparées entre les PVVIH sous TAR et les PVVIH naïfs de TAR. Les femmes sous TAR étaient significativement majoritaires à 72% (n=231) que les femmes naïves de TAR (p=0,001); le stade 3 de l´OMS était significativement prédominant chez les PVVIH sous TAR.

**Tableau 1:** caractéristiques générales de la population d’étude

Variables	PVVIH N (%) N=334 (100)	PVVIH sous TAR n (%) n=321 (96,1)	PVVIH sans TAR n (%) n=13 (13,9)	P-value
**Sexe**				**0,001**
Masculin	99(29,6)	90(28)	9(69,2)	
Féminin	235(70,4)	231(72)	4(30,8)	
**Age moyen (ans)**		51,06±11	47,62±13,59	0,290
**Stades OMS**				0,007
1	93	90(28)	3(23,1)	
2	78	77(24)	1(7,7)	
3	103	101(31,5)	2(15,4)	
4	60	53(16,5)	7(53,8)	
**Traitement antirétroviral**				
DTG		76(23,6)		
EFV		224(69,8)		
LPV/r		14(4,4)		
NVP		7(2,2)		

PVVIH: personne vivant avec le VIH; OMS: Organisation Mondiale de la Santé; TAR: traitement antirétroviral; DTG: dolutégravir; EFV: efavirenz; LPV/r: lopinavir/ritonavir; NVP: névirapine

**Comparaisons à travers les régimes des traitements antirétroviraux (TAR)**

**Selon les variables traditionnelles:** excepté la pression artérielle diastolique (P>0,05), il existait une variation inégale et très significative (P=0,000) des valeurs moyennes de l´âge, de pression artérielle systolique (PAS), de pression pulsée (PP), de tour de taille, de tour de hanche, de Tour de Taille/Tour de Hanche (TT/TH), de l´indice de masse corporelle (IMC), de glycémie, de low density lipoprotein-cholesterol (LDL-c), de high density lipoprotein (HDL-c), de Triglycérides (TG) et de cholestérol total (CT) à partir de DTG au LPV/r (Tableau 2).

**Tableau 2:** comparaisons des valeurs moyennes des variables traditionnelles de l’athérosclérose infraclinique à travers les groupes de TAR

Caractéristiques statistiques/variables indépendantes	DTG+ autres TAR	EFV+ autres TAR	NVP+ autres TAR	LPV/r+ autres TAR	ANOVA P-value
Age (ans)	46±10,3	52,9±10,6	68±9,4	75,3±0,8	<0,0001
PAS (mm Hg)	124,3±19,3	131±19,9	131,3±14,1	177,9±13,1	<0,0001
PAD (mm Hg)	78,5±12,2	79,6±12,1	73,7±6,5	78,2±17,5	0,565
PP (mm Hg)	45,4±13,6	51±16,2	57,5±15,8	99,7±15,8	<0,0001
Tour de Taille (Cm)	86,2±13,6	89,2±12,2	115,7±10,7	130,2±4,9	<0,0001
Tour de hanche (Cm)	97,5±14,1	101,5±11	114,8±11,5	128,3±10,2	<0,0001
Tour de Taille/Tour de Hanche	0,9±0,7	0,9±0,7	1,01±0,12	1,02±0,11	<0,0001
IMC (Kg/m2)	24,3±4,9	25,5±5,1	34,4±2,6	34,4±2,8	<0,0001
Glycémie (m/dL)	94,3±26,3	91,6±20,6	115,6±7,7	177,7±78,6	<0,0001
LDL-c (mg/dL)	107,2±37	92,7±33,5	58,4±13,5	36±14,4(S.E)	<0,0001
HDL-c (mg/dL)	47,3±6,3	34,5±16,2	15,5±6	8,1±1,7	<0,0001
TG (mg/dL)	92,8±34,5	84±33,8	69,7±25,4	19,4±4,8	<0,0001
Cholestérol total (mg/dL)	155,6±15,6	200,3±58,4	243,4±67	279,5±70,2	<0,0001

DTG: dolutégravir; EFV: efavirenz; NVP: névirapine; LPV/r: lopinavir/ritonavir; PAS: pression artérielle systolique; PAD: pression artérielle diastolique; PP: pression pulsée; IMC: indice de masse corporelle; LDL-c: low density lipoprotein- cholesterol; HDL-c: high density lipoprotein-cholesterol; TG: triglycérides

**Selon les variables émergentes:** excepté pour LDL-c/HDL-c (P>0,05), il existait une variation inégale et très significative (P=0,000) des valeurs moyennes de créatinine, d´acide urique, de protéine C-réactive (CRP), d´Index des pressions systoliques (IPS), d´Epaisseur intima-media carotidien (EIMc), de ratio Triglycérides/glucose (TyG), de non HDL-c, de ratio Triglycérides/HDL-c(TG/HDL) et de ratio cholésterol total/HDL-c(CT/HDL-c à partir de DTG au LPV/r (Tableau 3).

**Tableau 3:** comparaisons des valeurs moyennes des variables émergentes de l’athérosclérose infraclinique à travers les groupes de TAR

Caractéristiques statistiques/variables indépendantes	DTG+ autres TAR	EFV+ autres TAR	NVP+ autres TAR	LPV/r+ autres TAR	ANOVA P-value
Créatinine (md/dL)	0,94±0,21	1,02±0,26	1,37±0,77	3,12±1,44	<0,0001
Acide urique (mg/dL)	5,6±1,7	6,4±2,2	5,9±2,2	9±3,5	<0,0001
CRP (mg/L)	2,2±0,4	8,3±7,6	65,8±12,2	72,1±1,5	<0,0001
IPS	0,99±0,1	1,01±0,2	1,23±0,3	1,49±0,1	<0,0001
EIMC moyen (mm)	1,14±0,24	2,2±1,2	3,07±1,2	3,91±0,72	<0,0001
TyG après	4,5±0,27	4,5±0,26	4,54±0,20	4,9±0,45	<0,0001
TyG avant	9,29±0,27	9,9±0,26	10,24±0,20	10,8±0,45	<0,0001
Non HDL-c (mg/dL)	110,6±17,7	143±51,7	145,6±38	284,9±79,7	<0 ,0001
TG/HDL-c	1,95±0,79	2,30±1,06	3,11±1,20	2,31±0,31	0,007
LDL-c/HDL-c	2,25±0,79	2,43±0 ,81	2,58±0,58	2,65±12,7	0,179
CT/HDL-c	3,32±0,56	5,24±2,75	7,47±1,79	36,58±12,7	<0,0001
Acide urique/HDL-c (%)	11,6±0,04	36,4±32,1	76,7±24,9	154,2±53,8	<0,0001

DTG: dolutégravir; EFV: efavirenz; NVP: névirapin; LPV/r: lopinavir/ritonavir; CRP: protéine C réactive; IPS: index des pressions systoliques; EIMc: epaisseur intima-media carotidien; TyG: triglycéride/glucose; non HDL-c: non high density lipoprotein-cholesterol; LDL-c/HDL-c: low density lipoprotein cholesterol/high density lipoprotein-cholesterol; CT/HDL-c: cholestérol total/high density lipoprotein-cholesterol

**Comparaisons selon le traitement antirétroviral avant 2019 sans dolutégravir (DTG) et après 2019 avec dolutégravir**

**Selon les variables traditionnelles de l´athérosclérose infraclinique:** exceptées pour la pression artérielle diastolique (PAD) et la glycémie (P>0,05), les valeurs moyennes de l´âge, de pression artérielle systolique (PAS), de pression pulsée (PP), de tour de taille, de tour de hanche, d´Indice de masse corporelle(IMC) et de cholestérol total étaient significativement (P<0,05) supérieures dans l´ancien régime TAR sans DTG que dans le nouveau régime avec DTG. Par contre les valeurs de LDL-c, HDL-c et des triglycérides étaient plus élevées du côté du nouveau régime avec DTG (Tableau 4).

**Tableau 4:** comparaisons des biomarqueurs traditionnels athérogènes entre l’ancien régime TAR sans DTG et le nouveau régime TAR avec DTG

Caractéristiques statistiques/variables indépendantes	Ancien régime TAR sans DTG	Nouveau régime avec DTG	P-value
Age (ans)	54,6±11,7	46±10,3	<0,0001
Pression artérielle systolique (mm Hg)	133,4±22,3	124,3±19,3	0,001
Pression artérielle diastolique (mm Hg)	79,4±12,3	78,6±2,2	0,589
Pression Pulsée (mm Hg)	54±19,7	45,8±13,7	<0,001
Tour de Taille (Cm)	92,2±15,6	85,7±12,9	0,001
Tour de hanche (Cm)	103,6±12,7	99,2±13	0,009
Tour de Taille/Tour de Hanche (Cm)	0,9±0,08	0,9±0,06	0,026
Indice de masse corporel (Kg/m^2^)	26,2±5,4	24,4±4,9	<0,01
Glycémie (m/dL)	94,1±22	94,3±26,4	0,950
LDL-c (mg/dL)	88,5±37,3	107,2±37	<0,0001
HDL-c (mg/dL)	37,7±15,1	48,3±6	<0,0001
Triglycérides (mg/dL)	79,9±36	93,4±36	0,005
Cholestérol total (mg/dL)	206±62,1	155,6±15,5	<0,0001

DTG: dolutégravir; TAR: traitement antiretroviral; LDL-c: low density lipoprotein- cholesterol; HDL-c: high density lipoprotein-cholesterol

**Selon les variables émergentes de l´athérosclérose infraclinique:** les valeurs moyennes des marqueurs émergents de l´athérosclérose infraclinique ont été comparées entre l´ancien régime sans dolutégravir et le nouveau régime avec dolutégravir. Les valeurs moyennes de créatinine, d´acide urique, de protéine C-réactive (CRP), d´Index des pressions systoliques (IPS), d´épaisseur intima-media carotidien (EIMc), de charge virale, de ratio Triglycérides/glucose (TyG), de non high density lipoprotein-cholesterol (HDL-c), de ratio low density lipoprotein-cholesterol (LDL-c/HDL-c), de ratio cholestérol total/high density lipoprotein-cholesterol (CT/HDL-c), acide urique/HDL-c et de la durée du traitement étaient significativement (P<0,05) supérieures dans l´ancien régime TAR sans DTG que dans le nouveau régime avec DTG (Tableau 5).

**Tableau 5:** comparaisons des marqueurs émergents athérogènes entre l’ancien régime TAR sans DTG et le nouveau régime TAR avec DTG

Caractéristiques statistiques/variables indépendantes	Ancien régime TAR sans DTG	Nouveau régime avec DTG	P-value
Créatinine (md/dL)	2,2±0,8	0,9±0,2	<0,0001
Acide urique (mg/dL)	6,6±2,4	5,6±1,7	<0,001
CRP (mg/L)	13,9±1,3(S.E)	2,2±0,4	<0,0001
IPS	1,05±0,2	0,99±0,07	0,016
EIMC moyen (mm)	2,4±1,2	1,1±0,2	<0,0001
Charge virale (copies/ml)	8084,7±2053,2(S.E)	54,6±24,5(S.E)	0,030
TyG après	4,4±0,3	4,4±0,3	0,011
TyG avant	9,9±0,4	9,3±0,3	<0,0001
Non HDL-c (mg/dL)	173,6±68,4	108,4±17	<0,0001
TG/HDL-c	3,1±2	2±0,9	<0,0001
LDL-c/HDL-c	3,4±2,4	2,3±0,9	<0,0001
CT/HDL-c	9,8±0,6(SE)	3,4±0,6(S.E)	<0,0001
Acide urique/HDL-c (%)	44,3±43,5	11,7±3,8	<0,0001
Durée du traitement (ans)	10,9±7,5	2,4±1,1	<0,0001

DTG: dolutégravir; CRP: protéine C-réactive; IPS: index des pressions systoliques; EIMc: epaisseur intima-media carotidien; TyG: triglycéride/glucose; non HDL-c: non high density lipoprotein-cholesterol; LDL-c/HDL-c: low density lipoprotein cholesterol/high density lipoprotein-cholesterol; CT/HDL-c: cholestérol total/high density lipoprotein-cholesterol

**Déterminants indépendants du risque cardiométabolique traditionnel et émergent de l´athérosclérose infraclinique chez les patients vivants avec le VIH (PVVIH) sous traitement antirétroviral (TAR):** en considérant tous les biomarqueurs traditionnels et émergeants du risque cardiovasculaire significatif en analyse univarié, seuls les mariés (ORa: 4, IC95% 1,5-10,5; p<0,006), le niveau socioéconomique bas (ORa: 10,7, IC95% 2,3-48,7 p<0,002), la durée de l´infection par le VIH (ORa: 6,6, IC95% 2,8-16; p<0,0001), la durée du traitement antirétroviral ≥9 années (ORa: 0,3, IC 95% 0,2-0,7; p< 0,005) et le ratio cholestérol total /high density lipoprotein-cholesterol (CT/HDL-c)(ORa: 2, IC 95% 1,1-3,6; p=0,034) ont été retenus comme biomarqueurs déterminants indépendants dans l´analyse multivariée de type régression logistique binaire (Tableau 6).

**Tableau 6:** déterminants indépendants du risque cardiométabolique de l’ensemble de l’athérosclérose infraclinique dans la population des personnes vivant avec le VIH sous traitement antiretroviral

Variable	OR IC95%	P value	ORaj IC 95%	P-value
**Sexe**				
M	1,2(0,7-2)	0,303		
F	1			
**Age (ans)**				
≥60		0,001		
43-59				
<43				
**Etat civil**				
Marié	10(4,2-23,7)	<0,0001	4(1,5-10,5)	0,006
Célibataire	1		1	
**Niveau socio-économique**				
Bas	18,4(4,4-77,1)	<0,0001	10,7(2,3-48,7)	0,002
Elevé	1		1	
**Durée de l’infection (années)**				
≥14	7,6(3,8-15)	<0,0001	6,6(2,8-16)	<0,0001
8-13	5,5(2,8-10)		4,1(1,9-9,2)	0,001
<8	1		1	
**Durée du traitement (années)**				
≥9		0,013	0,3(0,2-0,7)	0,005
5-8			0,7(0,3-1,5)	0,248
<5			1	
**Tour de taille (Cm)**				
≥85	1,8(1,1-2,9)	0,012		
<85	1			
**Tour de Hanche (Cm)**				
≥100	1,7(1-3)	0,017		
<100	1			
**Indice de masse corporel (Kg/m^2^)**				
≥25	1,6(1-2,6)	0,024		
<25	1			
**CRP (mg/L)**				
Haut≥3	3(1,9-4,9)	<0,0001		
Bas <3	1			
**Créatinine (mg/dL)**				
≥1,5	3(1,8-5)	<0,0001		
<1,5	1			
**TG/HDL-c**				
≥2,2	2(1,3-3)	0,002		
<2,2	1			
**LDL-c/HDL-c**				
≥2,52	1,5(0,9-2,3)	0,07		
<2,52	1			
**CT/HDL-c**				
Haut ≥4	4(2,3-7)	<0,0001	2(1,1-3,6)	0,034
Bas <4	1	0,002		

CRP: protéine C-réactve; triglycérides/HDL-c; LDL-c/HDL-c: low density lipoprotein cholesterol/high density lipoprotein-cholesterol; CT/HDL-c: cholestérol total/high density lipoprotein-cholesterol

## Discussion

La présente étude a identifié les biomarqueurs du risque cardiométabolique capable de démontrer l´effet bénéfique du dolutégravir (DTG) par rapport aux autres antirétroviraux en milieu hospitalier de Kinshasa dans la prédiction de l´athérosclérose infraclinique. La population de cette étude était caractérisée par un âge avancé. L´âge moyen était de 51±12 ans. On observe une sénilité des PVVIH (stress oxydatif) lié à la contamination au-delà de 50 ans mais aussi au traitement antirétroviral qui maintient les patients longtemps en vie et en bonne santé [29,30]. Le sexe féminin était 2 fois plus rencontré dans la population d´étude. Soit 29,6% (n=99) hommes contre 70,4% (n=235) femmes. Cette féminisation de l´infection par le VIH est observée dans tous les pays et de façon plus marquée dans ceux où la transmission hétérosexuelle est très prédominante notamment en Afrique Subsaharienne [31]. La majorité (69,8%) (n=224) des PVVIH étaient sous EFV. Le choix thérapeutique initial est une décision essentielle pour l´avenir thérapeutique du patient. Le choix de la combinaison thérapeutique initiale tient souvent compte de plusieurs facteurs notamment de la disponibilité des molécules, le type de protocole adopté par chaque pays et de la situation particulière de chaque patient: l´existence d´anomalies biologiques, les traitements antituberculeux ou d´autres thérapies susceptibles d´interférer avec les ARV [32].

Excepté la PAD et le LDL-c/HDL-c, tous les marqueurs traditionnels et émergents de cette étude étaient élevés dans le groupe LPV/r, sauf LDL-c, HDL-c et TG qui étaient plus élevés dans le groupe DTG, TG/HDL-c dans le groupe EFV. Les inhibiteurs de la protéase sont principalement impliqués dans le risque cardiovasculaire, par leur capacité à induire une dyslipidémie et une résistance à l'insuline [33]. Par contre les inhibiteurs de l'intégrase n'ont jusqu'à présent pas montré d'anomalies lipidiques cohérentes lorsqu'ils sont utilisés chez des patients naïfs d'antirétroviraux [34]. Il a été noté également dans la littérature que les inhibiteurs non nucléosidiques de la transcriptase inverse (INNTI) sont pourvoyeurs de dyslipidémie, notamment une hypercholestérolémie totale et une hypertriglycéridémie [35-37].

Exceptées pour la PAD et la glycémie (P>0,05), toutes les valeurs moyennes des facteurs de risque traditionnels et émergents ainsi que de la charge virale et la durée du traitement ont été significativement supérieures dans l´ancien régime que dans le nouveau régime. En effet, il existe une relation entre les antirétroviraux et la présence d´un niveau de risque cardiovasculaire majeur. L´exposition à l´association lopinavir/ritonavir (inhibiteurs de la protéase) est un facteur augmentant ce risque. Marrakchi *et al*. [38] avaient fait le même constat. Blümer *et al*. [39] ont montré que les inhibiteurs de la protéase n´augmentaient le risque de survenue de l´insulinorésistance et éventuellement le risque d´évènement cardiovasculaire, que s´ils étaient associés à la zidovudine et à la lamivudine. Plusieurs études ont retrouvé des liens entre l´exposition aux différentes classes d´antirétroviraux et le risque cardiovasculaire [16,39-41].

Par contre, la dyslipidémie dans cette étude a été fréquente chez les patients sous DTG. Plusieurs études ont démontré le contraire [34,42,43]. Deux études randomisées réalisées en Afrique, l´essai NAMSAL [42] et l´essai ADVANCE [43] ont rapporté que les personnes prenant un traitement à base de dolutégravir, et principalement les femmes, connaissaient un gain de poids notable. Une nouvelle étude, basée sur une cohorte provenant de 12 sites, AFRICOS, soutenue par le PEPFAR (le programme présidentiel américain de lutte contre le VIH/sida) au Kenya, au Nigeria, en Tanzanie et en Ouganda confirment qu´un traitement basé sur le dolutégravir fait courir un risque non négligeable de surpoids aux patients [43]. La suppression de la charge virale était significativement dans le bras DTG. Plusieurs études l´ont également confirmé [34,42-44]. La durée d´exposition aux ARV supérieure ou égale à 9 ans (p = 0,013) était significativement associée à la présence d´athérosclérose comme dans l´étude de Aboubakar AM en Côte d´Ivoire [9].

Certaines études récentes rapportent que les nouveaux ARV diminuent la prévalence de la résistance à l'insuline chez les patients infectés par le VIH par rapport aux plus anciens ARV [3-5,45]. La durée d´exposition aux ARV est un facteur déterminant de l´athérosclérose [9]. Par contre la durée du DTG est courte par rapport aux autres ARV (effet déterministe et cumulatif). Seuls les mariés, le niveau socioéconomique bas, la durée de l´infection, la durée du traitement antirétroviral au-delà de 9 ans et le ratio CT/HDL-c ont été retenus comme déterminants indépendants d´athérosclérose en analyse multivariée type regression logistique. Par contre Aboubakar *et al*. en Côte d´Ivoire [9] ont trouvé la durée d´exposition aux ARV, l´âge et l´HTA comme déterminants de l´athérosclérose chez les PVVIH sous TAR. Kamdem *et al*. au Cameron [6] ont identifié tabagisme, le diabète et les dyslipidémies.

Malgré l'existence d'un traitement très efficace permettant aux personnes vivant avec le virus de l'immunodéficience humaine (PVVIH) de mener une vie relativement normale, l'inflammation chronique persiste et est à l'origine du développement prématuré de comorbidités liées à l'âge, telles que les maladies cardiovasculaires (MCV) comme l'athérosclérose. Le niveau socio-économique bas, non seulement limite l´accès à l´instruction, aux soins et à la formation sur les moyens de prévention contre les maladies transmissibles [46] mais aussi augmente le risque d´adoption de comportement sexuel à risqué [46,47]. Le ratio CT/HDL-c a été suggéré comme un risque athérogène statistiquement important [24,25].

Les résultats obtenus ne peuvent pas être généralisés à tous les hôpitaux de la R.D. Congo et le caractère hospitalier de l´étude ne permet pas de généraliser les conclusions à toute la population congolaise en général. Ce qui constitue les limites de notre étude. Par contre, la présente étude a le mérite d´avoir identifié les déterminants de l´athérosclérose infra clinique chez les PVVIH sous TAR sans DTG, une dyslipidémie sous le régime avec le dolutégravir et d´avoir utilisé la mesure de pression pulsée, de l´IPS et de l´EIMc chez les PVVIH à Kinshasa. Il serait nécessaire de faire un dépistage précoce de l´athérosclérose infraclinique chez les PVVIH naïves et celles sous TAR; de réaliser des études pour déterminer le rôle du DTG dans la dyslipidémie chez les adultes.

## Conclusion

Dans cette étude, la majorité des facteurs risque cardiométabolique traditionnels et émergents et l´échec virologique ont été retrouvés chez les PVVIH sous ancien régime de TAR sans DTG. La dyslipidémie a été fréquente du côté de DTG. Les mariés, le niveau socioéconomique bas, la durée de l´infection par le VIH, la durée du traitement antirétroviral au-delà de 9 ans et le ratio CT/HDL-c ont été identifiés comme déterminants de l´athérosclérose infraclinique chez les PVVIH sous TAR.

### Etat des connaissances sur le sujet

- *Les facteurs de risque traditionnels et émergents de maladies cardiovasculaires et les facteurs liés au VIH sont associés à l´athérosclérose subclinique chez les personnes infectées par le VIH dans la plupart des pays occidentaux;*
- *L'utilisation des antirétroviraux est associée à des effets métaboliques qui peuvent augmenter ce risque cardiovasculaire chez les PVVIH;*
- *Très utilisé actuellement, l´inhibiteur d'intégrase (INI: dolutégravir) induit une prise de poids dont les conséquences cardio-métaboliques sont encore mal connues*.

### Contribution de notre étude à la connaissance

- *L´étude a identifié les facteurs de risque cardiométabolique traditionnels et émergents chez les PVVIH en utilisant la mesure de la pression pulsée, l´index des pressions systoliques et l´Epaisseur Intima-média carotidien en milieu hospitalier de Kinshasa;*
- *Les déterminants de l´athérosclérose infraclinique trouvés dans cette étude sont fréquents chez les patients sous ancien régime de TAR sans DTG;*
- *La dyslipidémie est plus rencontrée chez les patients sous DTG*.
